# Special Issue: Emerging Dielectric, Piezoelectric, and Ferroelectric Ceramic and Crystalline Materials and Their Applications

**DOI:** 10.3390/ma15155118

**Published:** 2022-07-23

**Authors:** Irena Jankowska-Sumara, Magdalena Krupska-Klimczak

**Affiliations:** Institute of Physics, Pedagogical University of Krakow, 30-084 Kraków, Poland; magdalena.krupska-klimczak@up.krakow.pl

*Materials Physics*: Emerging Dielectric, Piezoelectric, and Ferroelectric Ceramic and Crystalline Materials and their Applications is an open Special Issue of *Materials*, which aims to publish original and review papers on new scientific and applied research and make great contributions to the identification and understanding of ceramic and crystalline materials which today enable applications that were previously virtually unimaginable. Dielectric materials are an integral element in all electronic circuits. In addition to their primary function of electrical isolation of circuit and device components, these materials also provide useful chemical and interfacial properties.

Dielectrics exhibit a full spectrum of electronic and structural properties, as well as a wealth of ferroic, piezoelectric, and other ordering phenomena. Such properties make many of these materials multifunctional and enable numerous device applications. Progress in the synthesis of dielectric systems—including crystals, ceramics, thin films, multilayers, and various nano-structures—and the advances in the fundamental understanding of their properties (structural, electronic, conductive) allow for conceptually new devices and systems with new functionalities or enhanced performance. At the same time, there are still many open questions and appealing possibilities regarding the engineering and application of the novel properties of these materials, as the field is becoming ever broader and moves forward in many different and exciting directions.

A special class of dielectrics is piezoelectrics. Piezoelectric elements have been used for many years in radio electronics and micro-electromechanical devices. Piezoelectric materials generate an electric charge when rapidly compressed by a mechanical force during vibration or motion, such as from machinery or an engine. The early piezoelectric devices were based on natural monocrystalline materials such as quartz or tourmaline and Rochelle salt; however, the effect was relatively small [1]. Nowadays, piezoelectrics are based mainly on polycrystalline ferroelectric ceramics such as barium titanate (BaTiO_3_) [2] and lead zirconate-titanate (PZT) [3,4], which exhibit larger displacements or induce larger electric voltages. Due to the adverse effects of lead on the environment, new effective lead-free piezoelectrics are still being sought out [5].

Ferroelectric materials are the next group of dielectric materials widely used in various devices such as memory elements, piezoelectric/electrostrictive transducers, actuators, pyroelectric infrared detectors, optical integrated circuits, and optical display devices. The switchable spontaneous polarization of ferroelectric materials confers upon them many useful properties with an extraordinarily wide range of applicability. In recent studies, it appears that ferroelectric materials are excellent candidates for modern cooling devices as electrocaloric materials. The practical use of pyroelectric and electrocaloric effects in devices continues to be of great interest. Experimental and theoretical studies on the response of ferroelectric domains to an external electric field and mechanical stress provide important information for applications of ferroelectric materials in energy storage and power-generation devices. In certain ferroelectric materials, the polarization is stable when aligned anti-parallel. Half of the dipoles in the material are oriented in one direction and their adjacent dipoles are oriented in the opposite direction such that there is no macroscopic net polarization in the material. This describes an antiferroelectric material. Solid solutions of antiferroelectrics with ferroelectrics also possess several useful properties. There are many reports that the most useful characteristics of these compounds reach the highest values in the region of phase transition, which are usually associated with structural instabilities. In this context, the study of the phase transition mechanisms in such materials is of high interest.

In this call, we welcome contributions in the field of the latest developments in advanced dielectric, piezoelectric and ferroelectric as well as antiferroelectric materials; high-strain, high-performance piezo-ceramics; novel processing; new materials; and novel properties of ferroelectrics, piezoelectrics, dielectrics, and related materials.
